# Inspired by struggle: A personal journey to global precision brain health

**DOI:** 10.1016/j.isci.2025.111918

**Published:** 2025-02-11

**Authors:** Agustin Ibanez

**Affiliations:** 1 Full Professor and Scientific Director of the Latin American Brain Health Institute (BrainLat), Universidad Adolfo Ibáñez, Santiago, Chile; 2 Professor in Global Brain Health, Global Brain Health Institute (GBHI), Trinity College Dublin, Dublin, Ireland

## Abstract

Dr. Agustin Ibanez joins iScience editor Maryam Fard to discuss his personal journey in brain health research. He advocates for integrating diversity in neurodegeneration studies, crucial for understanding conditions like dementia. He founded ReDLat and BrainLat, advancing research across Latin America and empowering underrepresented voices. This inspiring discussion showcases his commitment to fostering interdisciplinary approaches to drive global brain health progress. Dr. Ibanez is also the Guest Editor of iScience’s Special Issue: “Transdisciplinary approaches to arts and health: Integrating creative practice in clinical and public health contexts.”

**Figure:**
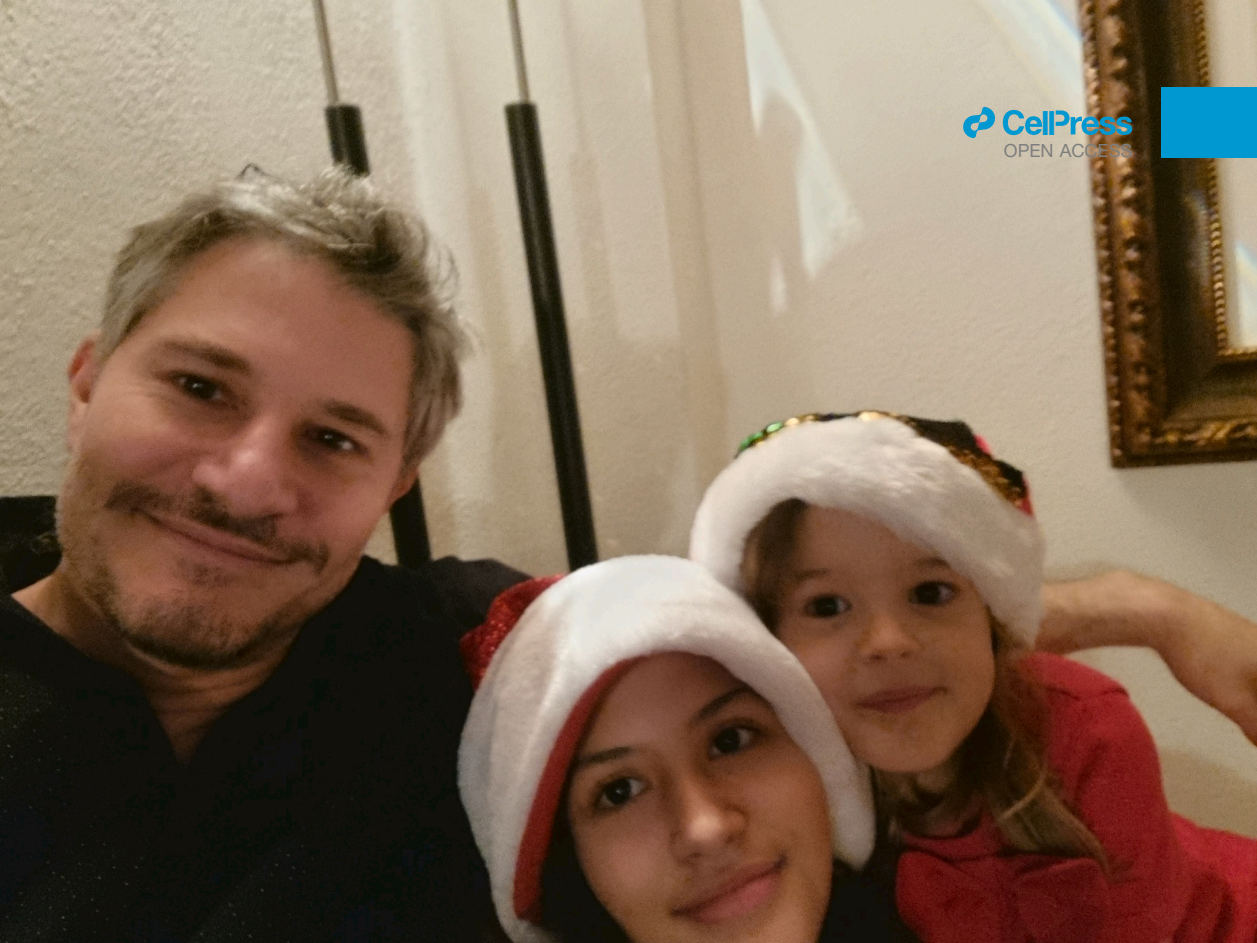
Above image: Agustin with his two daughters, Anahí (center) and Bianca (right)

Amid these difficulties, I found joy and resilience through my two daughters, Anahí and Bianca, who have deeply shaped me into a better person.

These experiences crystallized a single dream for me: to establish a regional consortium dedicated to expanding research and training in brain health, improving scientific capacity in the region, and bringing discoveries from the periphery to the forefront of global science.

Empowering local voices, data, and questions is not just a moral imperative; it’s a scientific requirement for advancing precision brain health science globally.

Diversity in research enhances our global understanding of dementia and ensures that interventions are more effective and equitable.

## Main text

### Inspired by struggle: A personal journey to global precision brain health

I was born in San Juan, Argentina, a semi-desert and isolated region where I grew up surrounded by mountains and nature. My early years were marked by struggles with attention-deficit/hyperactivity disorder and behavioral challenges in school, balanced by my love for playing guitar and compulsive reading. Through a series of unlikely circumstances, I became a neuroscientist, facing significant hurdles in accessing training and mentorship. My journey took me across Latin America, as well as Germany and the United States, where I pursued education and professional development despite ongoing struggles. My father developed a form of dementia that profoundly transformed his personality, strengths, and dreams. His illness also transformed me, leaving me profoundly frustrated both as a caregiver and as a researcher. Amid these difficulties, I found joy and resilience through my two daughters, Anahí and Bianca, who have deeply shaped me into a better person. Over time, I began collaborating with multiple labs across Latin America, where I repeatedly encountered the same systemic issues: limited funding and training opportunities, geographic isolation from mainstream science, and the sensation that our work lacked global relevance. These experiences crystallized a single dream for me: to establish a regional consortium dedicated to expanding research and training in brain health, improving scientific capacity in the region, and bringing discoveries from the periphery to the forefront of global science. I would have never imagined that, a few years later, that dream would come to life through ReDLat (the multi-partner consortium to expand dementia research in Latin America), an initiative that today boasts over 200+ publications, 90+ researchers, and 13 sites across the region.

### In your work, you emphasize the importance of empowering local voices. How do you see local research and cultural perspectives shaping global strategies for dementia prevention and healthy aging?

Working across Latin America, the US, Africa, Asia, and Europe has provided me with valuable insights into the challenges that arise even in the most well-intentioned and trustworthy research collaborations with underrepresented regions. These include patronizing attitudes, the discrediting of local ideas, the forced “fitting” of data patterns from minority groups into mainstream frameworks, the lack of confidence in disadvantaged research groups, the definition of research priorities by dominant groups, and a myriad of implicit biases that affect even the best-intentioned global collaborations. These challenges represent significant ethical barriers. But empowering local voices, data, and questions is not just a moral imperative; it’s a scientific requirement for advancing precision brain health science globally. Local research and cultural perspectives provide invaluable insights into the unique genetic, whole-body, environmental, and social factors that influence brain health in different populations. One of the most promising discoveries for advancing future protective effects against causative genes in dementia has been found in the global south, as well as many new variants not observed in other populations.^1^ Incorporating local voices allows us to move beyond one-size-fits-all models that often overlook the rich heterogeneity present in global populations. For instance, factors like socioeconomic status, education levels, cultural practices, and environmental exposures can significantly impact the onset, progression, and multimodal phenotypes of aging and dementia.^1^^,^^2^^,^^3^^,^^4^^,^^5^^,^^6^ In underrepresented populations, social and health disparities have larger effects than standard risk factors such as age or gender for healthy aging.^2^ Even universal models fail to be applied to underrepresented populations in terms of risk factors or brain-phenotype associations.^7^^,^^8^

Engaging local researchers beyond the US and Europe fosters innovation and scientific discovery.^1^ This diversity in research enhances our global understanding of dementia and ensures that interventions are more effective and equitable. My work at the Latin American Brain Health Institute (BrainLat) and the Global Brain health Institute (GBHI) has truly helped me co-build with a large community of hundreds of global fellows who are working to find universal approaches that foster equity, personalized, and precision brain health, while connecting the interests and needs of vulnerable communities and patients to research priorities. The development of local and regional initiatives has been very helpful in advancing a more mature science of brain health. I had the pleasure to found and co-direct ReDLat, an initiative with multiple centers across the Americas that began to evidence the impacts of genetics, epigenetics, and the physical and social exposome in the region.^3^^,^^4^^,^^5^

### What do you consider the most critical challenges in integrating diversity into dementia research, and how can we overcome these barriers to create more inclusive global brain health solutions?

One of the most critical challenges in integrating diversity into dementia and brain health research is the lack of theoretical and computational models that embrace the inherent complexity of brain health. Traditional models often focus on isolated factors—like genetics or brain imaging—without accounting for the holistic interplay between the brain, whole-body health, and environmental influences. This “standard” approach fails to capture the multifaceted nature of brain health, especially in diverse populations where these interactions can vary widely.

Another significant barrier is the misconception that simply correcting algorithmic biases or accumulating more data is sufficient. While these are important steps, they do not address the foundational issue: our models need to be fundamentally redesigned to incorporate complexity, multilevel integration, and heterogeneity. We also require transdisciplinary approaches that integrate insights from neuroscience, public health, social sciences, and computational modeling.

The universal attempt to provide a uniform global brain health framework is challenged by the large inequalities observed at the country level. Brain health is not the same across low, upper-middle, or high-income countries. It is not the same across the global north or south, or even the majority world. Of course, these distinctions are at some sense arbitrary.^1^ For instance, Chile is a high-income country, but less than 1% of patients with dementia can access gold standard imaging test (like positron emission tomography or PET, a medical scan that uses radioactive material to detect brain disorders). Argentina is an upper-middle-income country but has more than 50% child poverty. Australia is situated in the south but is not usually considered among the “global south” given the different socioeconomic conditions. And it is hard to imagine the US and Europe as a “world minority.” However, these distinctions only become a problem when we try to neglect the underlying diversity and differences between countries and regions in terms of brain health via universal approaches.

To overcome these barriers, brain health cannot be understood through simplistic, universal models. We need to develop new theoretical frameworks that can integrate brain metrics with multimodal data—including genetics, omics, whole-body health, and exposome factors like environmental and social influences.^9^ Investing in computational tools that can process and analyze this complex data is also essential. Advanced modeling techniques, such as generative biophysical modeling, multimodal and exposome-integration with brain metrics, and probabilistic metrics assigning individual trajectories through dynamic causal modeling, can help us understand how different factors interact at both the micro and macro levels.^10^ The models are more robust in integrating environmental factors (e.g., allostatic load^11^^,^^12^), body health interactions^10^ (e.g., physiological responses and anticipations to these environmental demands), as well as the neurodegenerative processes in different conditions such as frontotemporal dementia^11^ or Alzheimer’s disease and related conditions.^12^ Moreover, generative AI is opening incredible opportunities for scientists to become intellectual cyborgs,^13^ expanding predictions, models, and scientific programs. Fostering transdisciplinary collaboration will bring together experts from various fields to develop more holistic models.

### How do you define diversity? In healthcare, diversity is often limited to factors like sex and race; are there other important aspects that should be considered?

Multimodal diversity encompasses the heterogeneity observed across genetics, omics (such as proteomics and metabolomics), whole-body health, the exposome (which includes all physical and social exposures across a person’s lifetime), and multimodal brain measurements.^10^ Diversity, in the context of brain health, is a multifaceted concept that extends far beyond sex and race. Focusing solely on race or gender can neglect critical variables that significantly influence brain health. For example, socioeconomic status, cultural background, lifestyle choices, environmental exposures, and even geographical location can all impact the onset and progression of neurological conditions.^6^ These factors interact in complex ways, and their effects can vary dramatically between individuals and populations. My own perspective on diversity has evolved from trying to understand the embodied, embedded nature of cognitive neuroscience^14^ to incorporating the embodied (whole-body) and embedded (exposome) nature of brain health.^9^

**Figure:**
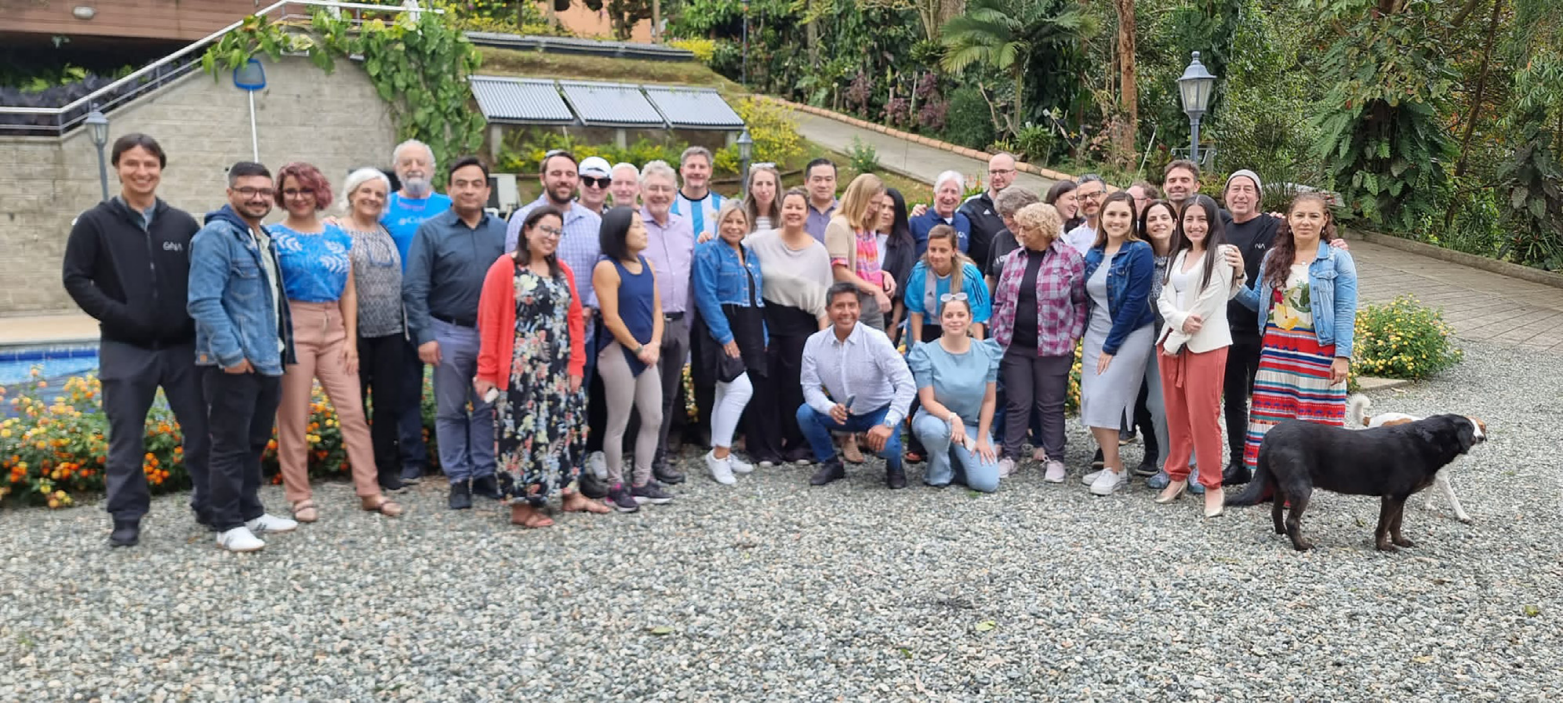
Annual meeting of ReDLat, hosted in the mountains of Medellín, Colombia, 2022

### Interdisciplinarity is a cornerstone of your approach. How does combining fields like neuroscience, public health, and social sciences enhance our ability to address complex issues in brain health?

Combining these fields is essential for effectively addressing complex issues in brain health. This interdisciplinary approach allows us to connect the macro-level societal and environmental factors with the micro-level processes occurring within the individual brain. By integrating these disciplines, we can develop comprehensive models that reflect the true complexity of brain health and disease. For instance, we used AI and multimodal neuroimaging (electroencephalogram [EEG] and fMRI) to develop measures of brain clocks and their brain age gaps (the difference between the estimated biological age of your brain and your chronological age). Using these brain clocks, we found that accelerated brain aging is influenced by multiple factors such as dementia and cognitive decline, gender disparities, chronic and non-chronic diseases, as well as physical (pollution) and social (structural socioeconomic inequalities) exposomes.^3^ Similarly, we showed that aggregate-level (city-level) inequality has stronger effects on brain health using both fMRI^8^ and EEG^5^ than individual factors such as gender, sex, education, or cognition, challenging conventional neuroscience approaches overemphasizing individual-level influences. To promote transdisciplinary dialogue, we have also developed brain health diplomacy toolkits^15^ for engaging with policymakers and health professionals without expertise in brain health. These findings inform policymakers to address disparities and environmental exposures, promoting equitable strategies for healthier brain aging across populations.

### What are the most important steps to ensure sustainable innovation and collaboration in brain health research, especially in regions like Latin America and the majority world?

To ensure sustainable innovation and collaboration in truly global brain health research, several key steps are necessary. We need new models of precision brain health that are tested in real-world settings and are complex enough to tackle inherent brain health complexities. Without such models, our understanding remains superficial, and interventions may not be always effective. Building bridges between scientists, policymakers, and stakeholders from both the public and private sectors is crucial. This collaboration ensures that research findings inform policy and that policies support scientific advancement. Blending local researchers and institutions with international initiatives enhances the ability to conduct relevant research and implement interventions tailored to specific community needs. These investments may lead to sustainable innovation driven by those who best understand the local context. Involving community members in the research process ensures that interventions are culturally sensitive and more likely to be accepted and sustained. We need to transform environments that support sustainable innovation and foster collaboration. In regions like Latin America, where unique challenges exist due to multimodal disparities, such interdisciplinary and collaborative efforts are particularly crucial.

## Acknowledgments

AI is supported by grants from the Multi-partner consortium to expand dementia research in Latin America [ReDLat, supported by 10.13039/100000061Fogarty International Center (FIC), 10.13039/100000002National Institutes of Health, 10.13039/100000049National Institute on Aging (R01 AG057234, R01 AG075775, R01 AG082056, R01 AG083799, CARDS-NIH Contract 75N95022C00031), 10.13039/100000957Alzheimer’s Association (SG-20-725707), 10.13039/100016608Rainwater Charitable Foundation – The Bluefield project to cure FTD, and Global Brain Health Institute)], ANID/10.13039/501100010751FONDECYT Regular (1210195 and 1210176 and 1220995); ANID/PIA/ANILLOS ACT210096; 10.13039/501100008736FONDEF ID20I10152, and ANID/10.13039/501100018735FONDAP 15150012. The contents of this publication are solely the responsibility of the authors and do not represent the official views of these institutions.
